# Overweight/obesity among adults in North-Western Ethiopia: a community-based cross sectional study

**DOI:** 10.1186/s13690-018-0262-8

**Published:** 2018-03-05

**Authors:** Teferi Mekonnen, Worku Animaw, Yeshaneh Seyum

**Affiliations:** 1 Bahir Dar University, School of Public Health, Public Health Nutrition Unit, Bahir Dar, Ethiopia; 2 Bahir Dar University, Department of Nursing, Bahir Dar, Ethiopia

**Keywords:** Overweight/obesity, Adults, Ethiopia

## Abstract

**Background:**

Nowadays adulthood overweight/obesity is an emerging public health problem in developing countries. There is no information on magnitude and contributing factors of adulthood overweight/obesity in Ethiopia, particularly in North West region of the country. Thus, the aim of this study was to assess magnitude and contributing factors of adulthood overweight/obesity in North West region of Ethiopia.

**Methods:**

A community-based cross-sectional study was conducted from September1, 2015 to November 30, 2015 in northwest region of Ethiopia particularly Bahir-Dar city and its rural districts. A total of 1484 adult participants were recruited in the study. Stratified multistage followed by systematic random sampling technique was employed to select participants. Overweight/obesity was determined using center for diseases control cutoff points. A multivariable binary logistic regression model was fitted to identify factors associated with overweight/obesity. Adjusted Odds Ratio (AOR) with the corresponding 95% Confidence Interval (CI) was calculated to show the strength of association.

**Results:**

A total of 1405 adults were participated in the study with a response rate of 94.7%. This study indicated that 11.3 (95% CI: 9.6, 13.1) adults were overweight and obese of which about 9.3% and 2% of adults were overweight and obese, respectively. The higher odds of being Overweight/Obese were noted among urban residents, females and older age. However performing mild to moderate physical activity [AOR = 0.608, 95% CI: 0.37, 0.99] and consumption of fruit and vegetable [AOR = 0.51, 95% CI: 0.34, 0.77] were found to be protective against overweight/obesity.

**Conclusion:**

Though, it was a problem of developed countries adulthood overweight /obesity is emerged as a public health problem among adults in Ethiopia particularly in the study area, northwest region. Hence, preventive interventions focusing on urban residents, females through encouraging Physical activity, fruit and vegetable consumption is essential to prevent emergence of adulthood overweight/obesity.

## Background

Overweight/Obesity is defined as the abnormal or excessive accumulation of body fat resulting from positive energy imbalance expressed by a body mass index (BMI) of 25–29.9 and ≥30 kg/m^2^, respectively [1, 2].

Overweight/Obesity have been increasing at an alarming rate in both the developed and developing countries during the last few decades, though it was historically a problem of the developed countries [3, 4].

In developing countries, along with economic development which leads to nutrition transition, numbers of people with overweight/obesity are increasing attributed by adopting a modern lifestyle with less physical activity and excessive consumption of energy-dense foods [5, 6].

Over the past 30 years, Overweight/obesity has been steadily increasing even in low-income countries, including those in Sub-Saharan Africa (SSA) [7, 8]. In Sub-Saharan African countries, overweight/obesity levels are still lower than in high-income countries but certainly higher than they were two decades ago and increasing at alarming rates [8, 9].

Demographic Health Survey (DHS) analysis of 32 Sub-Saharan African countries revealed that the pooled prevalence of overweight in the region was 15.9% with the lowest in Madagascar 5.6% and the highest in Swaziland 27.7%, Similarly, the prevalence of obesity was also lowest in Madagascar 1.1% and highest in Swaziland 23.0% [10]. Pocket studies in Tanzania revealed that the prevalence of overweight and obesity among adults were 24.1% and 19.2%, respectively [11]. Another study conducted in Malawi revealed that the prevalence of overweight and obesity were 20.7%, 7.4% respectively while overweight/obesity was 28.1% with the highest prevalence in urban areas that rural area [12].

Analysis of Ethiopian Demographic Health Survey (EDHS) found that the prevalence of overweight and obesity for urban settings were 12.1% and 2.8%,, respectively [13]. Another study in Addis Ababa found that 25.7% of women were overweight and 10.2% were obese. A study among permanent employees of the Commercial Bank of Ethiopia and teachers in government schools of Addis Ababa found that 24.7% of men and 25.7% of women were overweight and 2.1% men and 10.2 women were obese [9, 14, 15]. A study in Gondar revealed that the prevalence of overweight was 32.4% while the prevalence of obesity was 16.2% [16].

Different factors contribute to overweight and obesity among adults. Among the contributing factors identified, age [11, 17], sex [2, 11, 15, 18], educational status [11, 18–21], marital status [4, 11, 19, 20, 22, 23], occupational status [2, 23, 24], income [6, 11, 20, 25], residences [14, 17, 18, 26, 27], diet [19, 28], physical activity [2, 11, 23], alcohol consumption [4, 11, 23], and smoking [7, 9, 29, 30] were frequently reported in various studies.

In Ethiopia, particularly in the study area, there was no information regarding the prevalence and contributing factors of overweight/obesity among adults. Thus, this study determined the prevalence of overweight/obesity and associated factors among adults in the northwest region of Ethiopia. The information can be used as a baseline evidence for program planner, policymakers, researchers and organizations who are working on prevention of chronic non-communicable diseases.

## Methods

### Study design and period

A community-based quantitative cross-sectional study design was employed from 1st of September to 30th of November 2015 in north-western Ethiopia particularly Bahir-Dar city and its rural districts.

### Sample size and sampling procedure

The sample size was determined by using Epi Info™ 7 by considering the following assumptions, 28% prevalence of overweight EDHS 2011 result [13], design effect of 1.5, 15% non-response rate, 3% confidence limit and the final sample size was 1484. A systematic random sampling technique was used to select a total of 1484 adult participants.

### Study population

All adults who were living in the northwestern region of Ethiopia were targeted for the study. All adults aged ≥18 years who were living in selected districts during the data collection period were included in the study. Seriously ill adults during data collection were excluded from the study.

### Data collection tools and procedures

A structured pretested questionnaire was adapted from the WHO STEPwise questionnaire for Chronic Disease Risk Factor Surveillance was used in this study [31]. Data about dietary habit and physical activity were collected for a week duration based on the WHO STEPwise approach to chronic disease risk factor surveillance (STEPS) questionnaire.

The English version of the questionnaire was translated to the local language, Amharic, and back to English by a professional translator. Twenty four clinical diploma nurse data collectors and two BSc nurse supervisors were trained for data collection and supervision for two days. Finally, data were collected through interviewer-administered technique.

### Anthropometric measurements

The anthropometric assessment was done according to the standardized procedures stipulated by the Food and Nutrition Technical Assistance (FANTA) Anthropometric Indicators Measurement Guide [32]. Height was measured to the nearest 0.1 cm in standing position at Frankfurt plane with the occipital, shoulder and the buttock touches the vertical stand using a Stadiometer Seca (Germany). Weight was measured to the nearest 0.1 kg using an electronic weighing scale with wearing light clothes and with no shoes using Seca digital weighing scale. Finally, body mass index was computed.

### Data quality control

Training was given for data collectors and supervisors. The questionnaire was developed in English and then translated to Amharic and back to English then review was made for consistency of the translation. The data were collected after pre-testing the instrument. Regular supervision was made by supervisors. The collected data were reviewed and checked for completeness and accuracy by supervisors and investigator, and weight scale was calibrated and placed in levelled flat surface before each measurement was taken. Continuous checkups of the scale were carried out.

### Data processing and analysis

The data were coded on pre-arranged coding sheet by the principal investigator then entered into Epidata 3.1 and cleaned, processed and analyzed using SPSS version 20. The information obtained was described by using mean, frequencies, proportions, and tables. Body mass index was computed and Center for Diseases Control standard was used as a reference for classifying overweight/obesity.

Both bi-variable and multivariable logistic regression was used. Variables in the bi-variable logistic regression analysis having *P*-value of less than 0.2 were included in the multivariable logistic regression analysis to identify predictors that showed significant associations with overweight/obesity. After checking for multicollinearity enter method was used and Hosmer and Lemeshow goodness of model fit test was 0.88. Finally, the odds ratios with 95% confidence intervals were reported to indicate the strength of associations.

## Results

### Socio-demographic and economic characteristics

In this study, a total 1484 adult participants were recruited and 1405 of them participated in the study with a response rate of 94.7%. Of the total respondents, 56.7% were females, 65.8% were married and 65.1% were self-employed. With regard to educational status 37.4% were unable to read and write and of the total population included in the study, 50.8% were rural residents. The mean age of the participants were 37 years with standard deviation of 16. Majority of the respondents (91.6%) were Orthodox Christians. The households’ monthly mean income was 2251.55 ± 1245.757 Ethiopian Birr (Table 1).

**Table 1:** .Socio-demographic characteristics of adults in Northwestern Ethiopia, 2015 (*n*=1405)

Variable		Frequency	Percentage (%)
Sex	Male	609	43.3
	Female	796	56.7
Age	< 23 years	294	21.0
	24–28 years	270	19.3
	29–39 years	254	18.1
	37–50 years	312	22.3
	> 50 years	270	19.3
Religion	Orthodox	1283	91.6
	Muslim	97	6.9
	Protestant and others	20	1.4
Residence	Urban	691	49.2
	Rural	714	50.8
Occupational status	Student	110	7.9
	Formally Employed	161	11.5
	Self Employed	912	65.1
	Daily Laborer	217	15.5
Marital status	Single	319	22.8
	Married	922	65.9
	Divorced	72	5.1
	Widowed	87	6.2
Income	< 2250 Et.Birr	664	41.4
	> 2250 Et Birr	820	58.6

### Dietary habit and physical activity among adults residing in Northwest Ethiopia

About 843(60.2%) had consumed soft drinks always, 520(37.1%) had never consumed soft drinks and 37(2.6%) had sometimes consumed soft drinks. About 788(56.3%), 379(27.1%) and (233(16.6%) had consumed coffee always sometimes, and never respectively. About 350 (25%), 817(58.4%) and 233(16.6%) had always, sometimes and never used sugar added to food or drink respectively. About 73(5.2%) had ever chewed chat. About 60.9% had consumed fruit and vegetables and 745(53.2%) had performed mild to moderate physical activity (Table 2)**.**

**Table 2:** Dietary habit and physical activity of adults residing in Northwest region of Ethiopia, 2015 (*n* = 1405)

Variable		Frequency	Percentage (%)
Soft drink intake	Always	37	2.6
	Sometimes	843	60.2
	Never	520	37.1
Coffee drinking	Always	788	56.3
	Sometimes	379	27.1
	Never	233	16.6
Sugar added to any food /drinks	Always	350	25.0
	Sometimes	817	58.4
	Never	233	16.6
Ever chew chat	Yes	73	5.2
	No	1327	94.8
Fruit and vegetable intake	Yes	852	60.9
	No	655	46.8
Performing mild to moderate Physical activity	Yes	745	53.2
	No	655	46.8

### Anthropometric measurements and prevalence of overweight /obesity among adults in North West region of Ethiopia, 2015

The mean height ± SD was 162 ± 0.09 cm and the mean weight ± SD was 55 ± 9 kg.

The overall prevalence of overweight/obesity was 11.3 (95% CI: 9.6, 13.1), in which 9.3% and 2% of adults were overweight and obese respectively. The proportions of overweight/obesity among female and male were 15% and 6.4% respectively. Moreover 18.7% urban residents and 4.2% rural residents were overweight/ obese.

### Factors associated with overweight/obesity among adults in North West region of Ethiopia

The multivariable logistic regression analysis revealed that being female, being in an urban resident, performing mild to moderate physical activity; fruit and vegetable intake and age were significantly associated with adulthood overweight and obesity. Accordingly, the odds of being overweight /obese were 3.12 times higher in urban residents as compared to rural residents [AOR = 3.12, 95% CI: 1.76, 5.54]. Similarly, the higher odds of being overweight/obesity were noted among females as compared to males [AOR=, 2.72, CI (1.74, 4.23] and a higher odds of being overweight/obese were noted in the age category of 24–28 years [AOR = 2.82, 95% CI: 1.33,5.98], 29–36 years[AOR = 5.3, 95% CI: 2.43, 11.56],37–50 years [AOR = 6.06, 95% CI: 2.77, 13.29], greater than 50 years [AOR = 7.14, 95% CI: 3.23, 15.79] as compared with age category of 18–23 years old.

A lower odds of being overweight/obese were noted among those who performed mild to moderate physical activity and who had consumed fruits and vegetables. Those who had performed mild to moderate physical activity were 39.2% times less likely to develop overweight/obesity [AOR = 0.608, 95% CI: 0.37, 0.99] and those who had consumed fruits and vegetables were 49% times less likely to develop overweight obesity [AOR = 0.51, 95% CI: 0.34, 0.77] (Table 3) than their counterparts.

**Table 3:** Factors associated with overweight/obesity among adults in Northwestern region of Ethiopia, 2015 (*n* = 1405)

Variables		Overweight/obesity		COR (95% CI)	AOR (95% CI)
		Yes	No		
Sex	Male	39	570	1	
	Female	120	676	2.59 (1.78–3.79)	2.72 (1.74–4.23) **
Performing mild to moderate physical activity	Yes	43	705	0.28 (0.19–0.41)	0.61 (0.37–0.99) **
	No	116	541	1	
Residence	Rural	30	684	1	
	Urban	129	562	5.23 (3.46–7.91)	3.12 (1.76–5.54) **
Age category	< 23	14	183	1	
	24–28	25	246	2.05 (1.05–4.04)	2.82 (1.33–5.98**
	29–36	34	220	3.12 (1.64–5.97)	5.30 (2.43–11.56) **
	37–50	38	274	2.80 (1.49–5.29)	6.06 (2.77–13.29) **
	> 50	48	223	4.35 (2.34–8.09)	7.14 (3.23–15.79) **
Fruit and vegetable intake	Yes	40	509	0.49 (0.33–0.71	0.51 (0.34–0.77) **
	No	119	737	1	

*Note:* *p-value < 0.05, **p-value< 0.001, CI = Confidence Interval, COR = Crude Odds Ratio, AOR = Adjusted Odds Ratio

## Discussions

This study demonstrated that the overall prevalence of overweight/obesity among adults in northwestern region of Ethiopia was found to be 11.3% [95% CI: 9.6–13.1], of which 9.3% were overweight and 2.0% were obese. The finding of this study is consistent with the evidence from EDHS data analyzed for the urban setting where 12.1% and 2.8% were overweight and obese respectively [13].

The finding of the study is also lower than the pooled prevalence of Demographic Health Survey data of 32 sub-Saharan African countries yielding 15.9% prevalence of overweight in the region [10] and studies done in Malawi (28.1%) and Tanzania in which the prevalence of overweight and obesity among adults were 24.1% and 19.2% [33]. The discrepancy might be due to the disparities in socio-demographic characteristics and dietary habits.

In this study female adults were more likely to be overweight/ obese as compared to males. The finding is consistent with the study done in Ethiopia and Ghana[13, 34]. This might be due to fact that females can carry more amount of fat as compared to males.

Residency was found to be one of the factors associated with the nutritional statuses of the participants. A higher odds of being overweight/obese were found in urban residents as compared to the rural residents. This finding is consistent with the study done in Addis Ababa, Gondar, Benin and Chennai [14, 17, 18, 26, 28]. This might be due to the fact, in urban resident’s practice more sedentary lifestyle and faster nutrition transitions like focusing on synthetic and energy-dense foods.

A higher odds of being overweight/obese was noted in the age category of 24 years and above. This finding is consistent with the study done in Ghana and EDHS2011 finding [4, 13, 35, 36]. This might be due to the fact that as age increases the likelihood of sedentary life style would also increased [37, 38]. However, performing mild to moderate physical activity were found to be protective against overweight/obesity in which those who had performed mild to moderate physical activity were less likely to develop overweight/obesity as compared to those who did not. The finding of this study is consistent with the study done in Tanzania [33]. This might be due to the fact that physical activity determines the amount of calories either spent or stored in the body in the form of fat and maintains healthy weight status and because of its potential major impact on body composition, metabolism, and increasing energy expenditure.

Fruit and vegetable consumptions were found to be protective against overweight/obesity in which those who consumed fruit and vegetables were less likely to develop overweight/obesity. The finding of this study is consistent with the study done in Chennai [19]. This might be due to the fact that their bulk and low energy density of fruit and vegetable (with a high amount of water and fiber) are believed to reduce energy dense food consumption and helps to easily attain satiety.

### Limitation of study

Since it is cross-sectional study it is not far from the pitfalls of cross-sectional study. History of past diseases or present history of co-morbidity was not assessed. Frequency and portion size of fruit and vegetable consumptions, alcohol consumptions were not included in the final model due to issues of recall bias.

## Conclusion

In this study, the overall prevalence of overweight/obesity among adults in northwest region of Ethiopia was found to be 11.3%. The higher odds of being overweight /obese were noted among females, urban residents and older age participants. Whereas performing mild to moderate physical activity and consumption of fruits and vegetables were found to be protective against overweight/obesity. As this evidence shows overweight/obesity which was a problem of the developed country has now emerged in Ethiopia particularly in the study area, northwest region. Hence, preventive interventions by focusing on urban residents, females through encouraging physical activity and fruit and vegetable consumption is essential to prevent the emergence of adulthood overweight/obesity and hence could prevent its related health problems such as diabetes mellitus, hypertension, heart problems, stroke and many more.

## Abbreviations

AOR: Adjusted odds ratio
BMI: Body mass index
CI: Confidence interval
EDHS: Ethiopian Demographic Health Survey
WHO: World Health Organization
